# Hamp1 but not Hamp2 regulates ferroportin in fish with two functionally distinct hepcidin types

**DOI:** 10.1038/s41598-017-14933-5

**Published:** 2017-11-01

**Authors:** João V. Neves, Miguel F. Ramos, Ana C. Moreira, Tânia Silva, Maria S. Gomes, Pedro N. S. Rodrigues

**Affiliations:** 1 i3S - Instituto de Investigação e Inovação em Saúde, Universidade do Porto, Portugal, Rua Alfredo Allen 208, 4200-135 Porto, Portugal; 2 IBMC – Instituto de Biologia Molecular e Celular, Universidade do Porto, Rua Alfredo Allen 208, 4200-135 Porto, Portugal; 3 ICBAS - Instituto de Ciências Biomédicas Abel Salazar, Universidade do Porto, Rua de Jorge Viterbo Ferreira 228, 4050-313 Porto, Portugal

## Abstract

Hepcidin is a small cysteine rich peptide that regulates the sole known cellular iron exporter, ferroportin, effectively controlling iron metabolism. Contrary to humans, where a single hepcidin exists, many fish have two functionally distinct hepcidin types, despite having a single ferroportin gene. This raises the question of whether ferroportin is similarly regulated by the iron regulator Hamp1 and the antimicrobial Hamp2. In sea bass (*Dicentrarchus labrax*), iron overload prompted a downregulation of ferroportin, associated with an upregulation of *hamp1*, whereas an opposite response was observed during anemia, with no changes in *hamp2* in either situation. During infection, ferroportin expression decreased, indicating iron withholding to avoid microbial proliferation. *In vivo* administration of Hamp1 but not Hamp2 synthetic peptides caused significant reduction in ferroportin expression, indicating that in teleost fish with two hepcidin types, ferroportin activity is mediated through the iron-regulator Hamp1, and not through the dedicated antimicrobial Hamp2. Additionally, *in vitro* treatment of mouse macrophages with fish Hamp1 but not Hamp2 caused a decrease in ferroportin levels. These results raise questions on the evolution of hepcidin and ferroportin functional partnership and open new possibilities for the pharmaceutical use of selected fish Hamp2 hepcidins during infections, with no impact on iron homeostasis.

## Introduction

Hepcidin, a small cysteine rich peptide originally characterized as an antimicrobial peptide, is currently considered the key regulator of iron metabolism^1–3^. This role is exerted through the regulation of Ferroportin (FPN1), also known as iron-regulated gene 1 (IREG1), metal transporter protein 1 (MTP1), solute carrier family 11 member 3 (SLC11A3) or solute carrier family 40 member 1 (SLC40A1), a transmembrane protein that has been independently identified by three distinct research groups as the sole known cellular iron exporter^4–6^. Additionally, it is also able to transport zinc and cobalt, but not manganese, cadmium or copper, in their divalent states^7^.

Ferroportin plays a critical role in iron homeostasis, being expressed in all sites involved in iron export to the plasma, including the basolateral membranes of duodenal enterocytes (iron uptake and export into circulation)^4,6,8^, hepatocytes (iron mobilization from storage)^9^, reticuloendothelial macrophages (iron recycling from senescent red blood cells)^8^ and the embryonic syncytiotrophoblasts (placental iron transfer to the embryo)^6^. Its expression is regulated at several levels: transcriptional, post-transcriptional, and post-translational. At the cellular level, ferroportin expression is stimulated/repressed through the IRE/IRP regulatory system^5,10,11^, whereas at the systemic level, ferroportin is mostly regulated by hepcidin^9,12^.

Despite all that is known about hepcidin and ferroportin, their relation is still surrounded by several controversies. The mechanisms by which hepcidin regulates ferroportin are known to involve three fundamental steps, but are not yet fully understood. When iron levels are high or an infectious/inflammatory process occurs, hepcidin binds to ferroportin^12^, leading to its ubiquitination^13,14^ and degradation in late endosome/lysosome compartments^15^, blocking the release of iron^9,16^. During infection, this blockade of iron release not only limits iron availability for pathogens, but also for normal erythropoiesis, which if prolonged can lead to the condition referred as anemia of inflammation^17,18^. Contrary, when iron or oxygen levels are low, hepcidin production is attenuated or suppressed, increasing cellular iron efflux and availability for enhanced erythropoiesis and other metabolic processes.

There are also conflicting reports regarding several ferroportin features which could influence the way hepcidin binds to or blocks it, such as the number of transmembrane domains, which can range from 9 to 12, and consequently to the position of the COOH terminus, which may be either on the extracellular or cytoplasmic side of the membrane^4,6,19–24^. The oligomeric state of ferroportin is also under debate, with some studies suggesting a monomeric state^25–27^, while others suggest ferroportin as a dimer/multimer^28,29^. The most recent studies characterize ferroportin as a monomer, with 12 transmembrane domains and both termini in the cytoplasmic side^20^. In order to identify the region involved with hepcidin binding, several studies have analyzed the functional consequences of mutations in ferroportin, and depending on the amino acid substitution or the assay used, the results range from innocuous to resistance to hepcidin degradation or total loss of function^21,22,25,28,30–34^.

The ferroportin gene is highly conserved during evolution, being present from the simplest bacteria to the highest vertebrates. Since its discovery, most studies have focused on human ferroportin and associated diseases^20–22,35^, with more recent studies showing a renewed interest in bacterial ferroportin^19,36^. As such, information is relatively scarce regarding teleost fish, especially considering that its identification and earlier functional characterization was performed using zebrafish^6^. The study of the interaction between hepcidin and ferroportin in teleost fish is particularly interesting because, contrary to most mammals where a single hepcidin exists, many teleosts have two distinct functional hepcidin types, hamp1 and hamp2, with the former mostly involved in the regulation of iron metabolism and the later assuming an exclusively antimicrobial role^37–39^. This poses the question of whether different hepcidin types may have different effects on ferroportin activity. If this is the case, the potential use of fish-derived hepcidins for the differential treatment of iron related or infectious diseases can be envisioned, not only in fish, but in other species as well.

With the present work, we investigate the interaction between hepcidin and ferroportin in the European sea bass (*Dicentrarchus labrax*), a teleost fish that possesses two functional hepcidin types, previously described by our laboratory^37^. We characterize its ferroportin at the DNA and protein levels, assess its constitutive tissue transcription levels, evaluate variations in its expression during a variety of experimental conditions, including iron overload, anemia, and infection, and investigate the effects of the administration of hepcidin peptides in its expression.

## Results

### Molecular characterization of sea bass ferroportin

A single sea bass ferroportin transcript was obtained by PCR amplification and 5′/3′ RACE using liver and intestine cDNA. Ferroportin coding DNA (deposited in GenBank under accession number KU599935) consists of an open reading frame of 1707 bp (sup. Figure 1), with flanking 5′ and 3′ untranslated regions of 457 bp and 729 bp, respectively, and encodes a 568 aa protein. The peptide has a predicted m.w. of 62078 Da and an isoelectric point of 5.63. A single Iron Responsive Element (IRE) was predicted in the 5′ region using RegRNA 2.0^40^. The genomic structure of ferroportin consisted of eight exons and seven introns (sup. Table 1) (deposited in GenBank under accession number KU599934), with a single initiator methionine and stop codon. The genomic interval from the initiator methionine to the stop codon is 6010 bp. Genomic organization of sea bass ferroportin was analyzed and compared with those of the other animal species, including other fish, reptiles, amphibians, birds, mammals and invertebrates, and found to be very similar with the usual (with the exception of *C*. *elegans*) organization in eight exons and seven introns. Similarities in exon sizes were also observed for all vertebrates, but significant variations occur in intron sizes (sup. Table 1).

**Figure 1:**
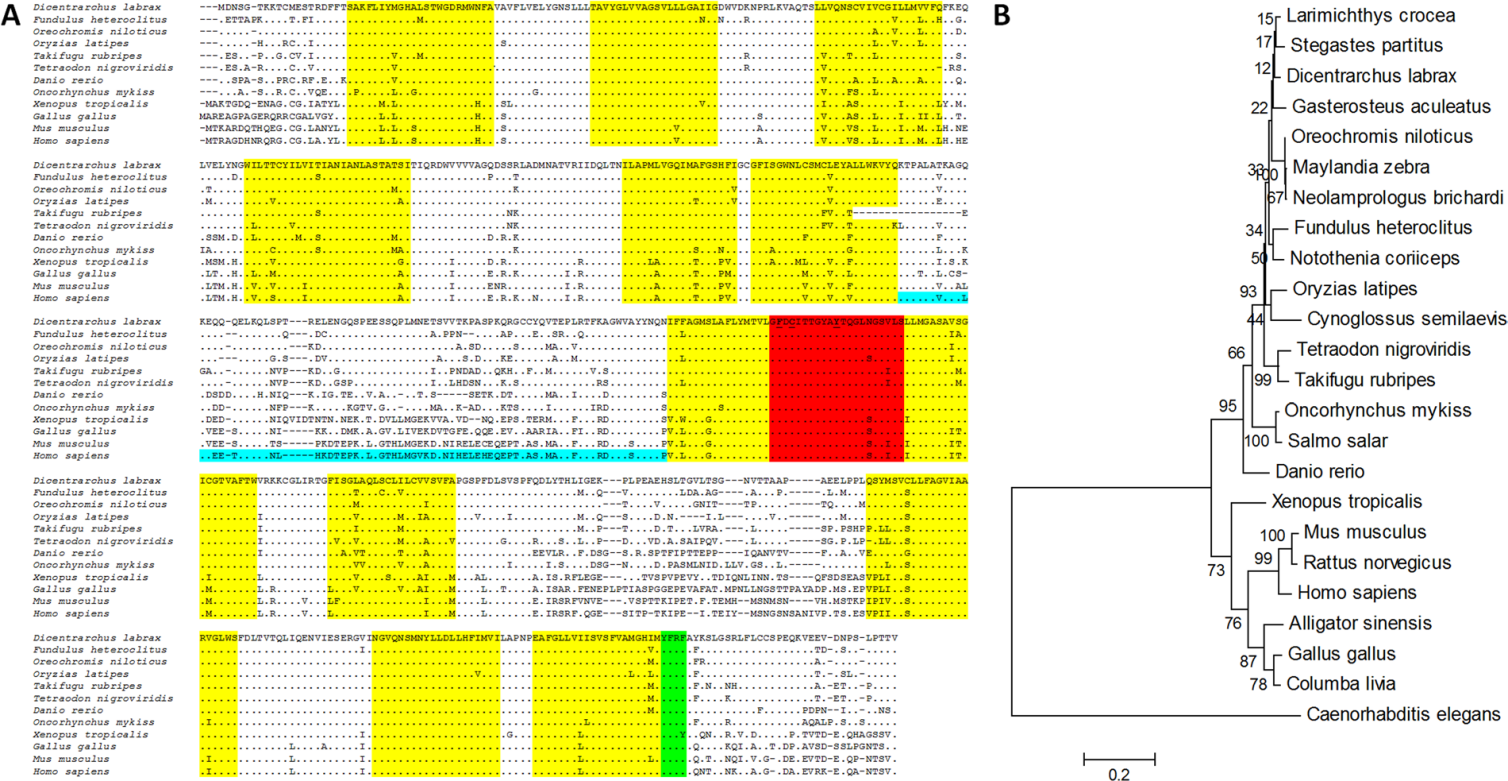
Amino acid alignment and phylogenetic analysis. (**A**) Sea bass ferroportin was aligned with ferroportins from other vertebrates. Identical residues and gaps are indicated by dots and dashes, respectively. Signature features and putative motifs are highlighted as follows: yellow, transmembrane domains^21,44^; red, extracellular hepcidin-binding loop^43,44^, with key residues required for hepcidin binding underlined; blue, cytoplasmic loop that undergoes hepcidin-dependent ubiquitination^14,44^; green, AP-2 adapter-binding motif for clathrin-dependent endocytosis^44^. (**B**) The evolutionary history was inferred by using the Maximum Likelihood method based on the JTT matrix-based model^69^. The bootstrap consensus tree inferred from 1000 replicates is taken to represent the evolutionary history of the taxa analyzed. Branches corresponding to partitions reproduced in less than 50% bootstrap replicates are collapsed. The percentage of replicate trees in which the associated taxa clustered together in the bootstrap test (10000 replicates) is shown next to the branches. Initial tree(s) for the heuristic search were obtained by applying the Neighbor-Joining method to a matrix of pairwise distances estimated using a JTT model. The analysis involved 24 amino acid sequences. All positions containing gaps and missing data were eliminated. There were a total of 453 positions in the final dataset. Evolutionary analyses were conducted in MEGA6^64^.

### Sequence comparison and phylogenetic analysis

Sequence comparison with other vertebrate and invertebrate species ferroportin proteins showed a high degree of conservation, sharing with them an identity between 30.45% (*C*. *elegans*) and 95.75% (*L*. *crocea*). The putative features observed for mammalian and bacterial ferroportins also seem to be present and relatively conserved in sea bass (Fig. 1A), namely twelve transmembrane domains, an extracellular hepcidin-binding loop with conserved key residues and the AP-2 adapter-binding motif for clathrin-dependent endocytosis. The less conserved feature is the cytoplasmic loop that undergoes hepcidin-dependent ubiquitination, which varies significantly among species but still maintaining some degree of conservation, particularly in the starting and ending portions. Additionally, almost all amino acid residues typically associated with pathogenic mutations in human ferroportin are also conserved in sea bass (sup. Figure 2). Phylogenetic analysis clusters fish ferroportins separated from most tetrapods (Fig. 1B). Among fish, there seem to be four separate clusters. One cluster includes a single member of the Cypriniformes order (*D*. *rerio*), a second cluster includes both Salmoniformes, a third cluster includes both Tetraodontiformes and the fourth cluster includes fish from the Perciformes, Pleuronectiformes, Beloniformes, Cyprinodontiformes and Gasterosteiformes orders.

### Molecular Modeling

**Figure 2:**
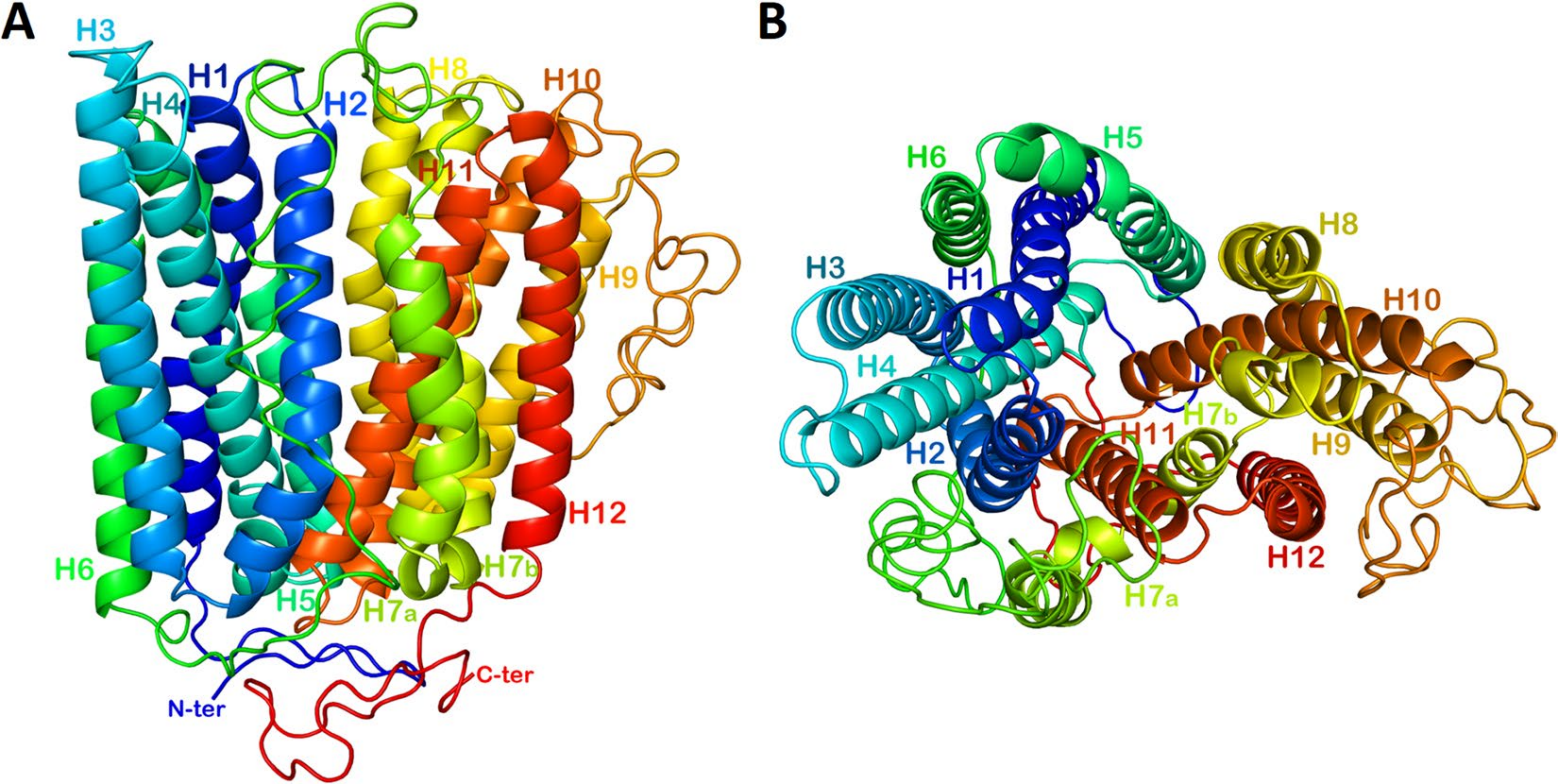
Theoretical model of sea bass ferroportin 3D structure. (**A**) side view from the membrane plane; (**B**) top view from the extracellular side. Ribbons are rainbow coloured from blue to red, starting from the N-terminus and ending in the C-terminus. The borders of the helices, as predicted by Phyre2, are as follows: H1: Ser20-Leu50; H2: Leu56-Asp81; H3: Arg85-Phe111; H4: Ile125-Ile151; H5: Ser165-Phe195; H6: His198-Gln236; H7: Gln280-Thr333; H8: Gly338-Lys365; H9: Ile369-Phe391; H10: Gln446-Glu478; H11: Glu482-Leu509; H12: Pro513-Ser541.

**Figure 3:**
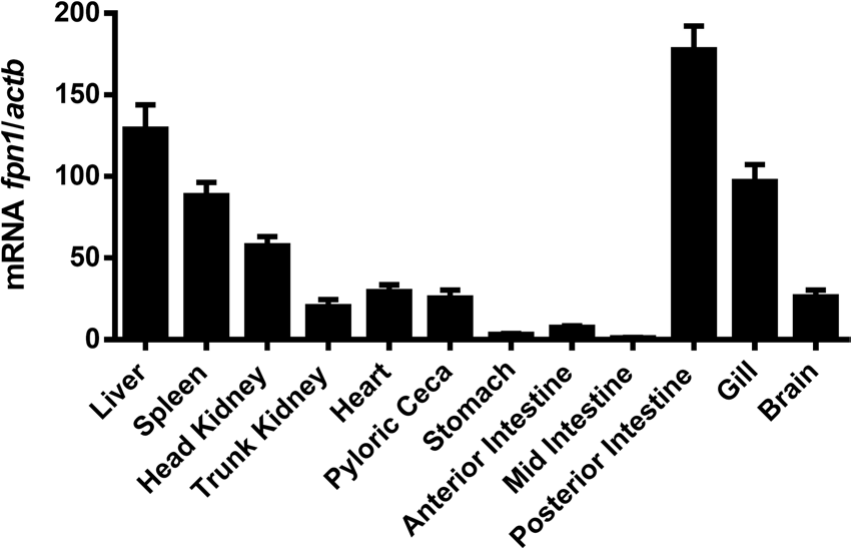
Constitutive expression of sea bass ferroportin. Expression was measured by real-time PCR in several organs of healthy sea bass specimens (n = 5). Values are expressed as mean fold change ± SD. *Actb* was used as the housekeeping gene.

A profile-profile alignment webserver (Phyre2) was used to search for sequence similarities between sea bass ferroportin and membrane proteins with known 3D structures that could serve as template(s) for homology modeling. Phyre2 showed two significant alignments with a confidence of 100% and two more with a confidence of 99.9%, with sequences of four Major Facilitator Superfamily (MFS) Transporters members whose 3D structures are presently known at an atomic resolution: (1) *Bdellovibrio bacteriovorus* ferroportin (FPN), pdb 5ayn^36^, E-value 1.2e-43, 23% identity; (2) *Bdellovibrio bacteriovorus* ferroportin (FPN), pdb 5aym^36^, E-value 1.2e-41, 24% identity; (3) *Escherichia coli* glycerol-3-phosphate transporter (GlpT) pdb 1pw4^41^, E-value 8.4e-17, 12% identity; (4) *Escherichia coli* multidrug transporter (Mdfa), pdb 4zp0^42^, E-value 6e-15, 11% identity. Two templates were selected to model the 3D structure of sea bass ferroportin (Fig. 3), based on heuristics to maximize confidence, percentage identity and alignment coverage: FPN (pdb 5ayn) and FPN (pdb 5aym). From a total of 568 residues, 403 (71%) were modeled at 100% confidence, whereas the remaining 165 (29%) were modeled *ab initio*. Secondary structure predictions reported by Phyre2 indicate the occurrence of 12 transmembrane helices (Fig. 2), predicted to form two similar domains comprised by 6 helices each and interconnected by a long cytoplasmic loop, and with both N- and C-termini being placed intracellularly.

### Constitutive expression of sea bass ferroportin

Ferroportin constitutive expression was evaluated in several healthy sea bass tissues, namely, liver, spleen, head and trunk kidney, heart, pyloric ceca, stomach, several sections of the intestine, gill and brain (Fig. 3). The highest expression was observed in the posterior intestine, followed by the liver, gill, spleen and head kidney; moderate in the trunk kidney, brain, heart and pyloric ceca; and low in the stomach and anterior and mid sections of the intestine.

### Hepcidin and ferroportin expression in *in vivo* experimental models

Sea bass *hamp1*, *hamp2* and *fpn1* mRNA expression levels were evaluated in response to an assortment of experimental conditions known to influence their expression. Tested conditions included iron overload, anemia and infection with several Gram-negative and Gram-positive bacteria.

**Figure 4:**
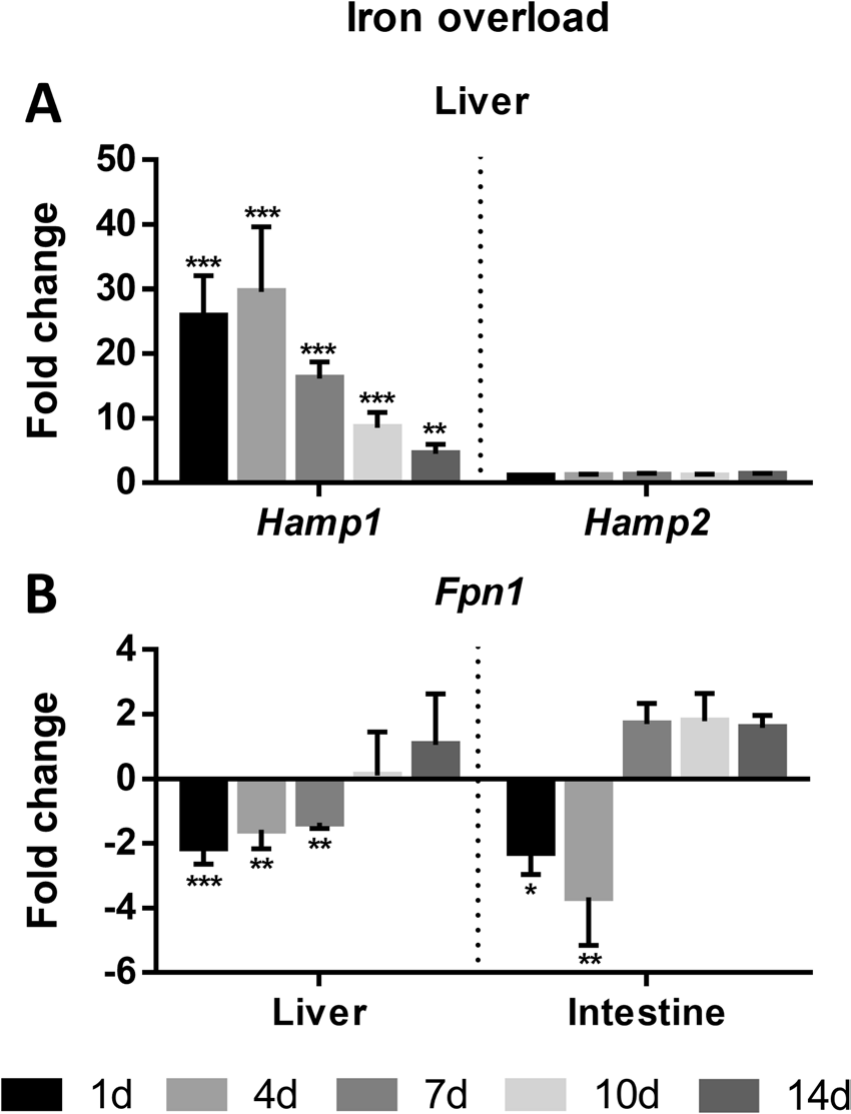
Sea bass (**A**) hepcidin expression in the liver and (**B**) ferroportin expression in the liver and intestine under experimental iron overload. Fish were injected with 2 mg of iron dextran (n = 4). Values are expressed as mean fold change ± SD. *Actb* was used as the housekeeping gene. Differences from the control groups were considered significant at *p < 0.05, **p < 0.01, and ***p < 0.001.

*Iron overload*. In the experimental model of iron overload, *hamp1* and *hamp2* expressions were evaluated in the liver (Fig. 4A), and *fpn1* expression was evaluated both in the liver and intestine (Fig. 4B), 1, 4, 7, 10 and 14 d after overload with 2 mg of i.p. injected iron dextran. *Hamp1* was significantly overexpressed throughout the duration of the experiment, starting to increase as early as day 1, reaching maximum overexpression at day 4 and gradually decreasing towards day 14, although still not recovering to control levels. *Hamp2* expression on the other hand, did not significantly change at any time point. In the liver, *fpn1* expression was significantly decreased up to day 7, recovering to control levels at day 10. In the intestine, significant decreases in expression were observed at days 1 and 4, with a recovery to control levels at day 7.

**Figure 5:**
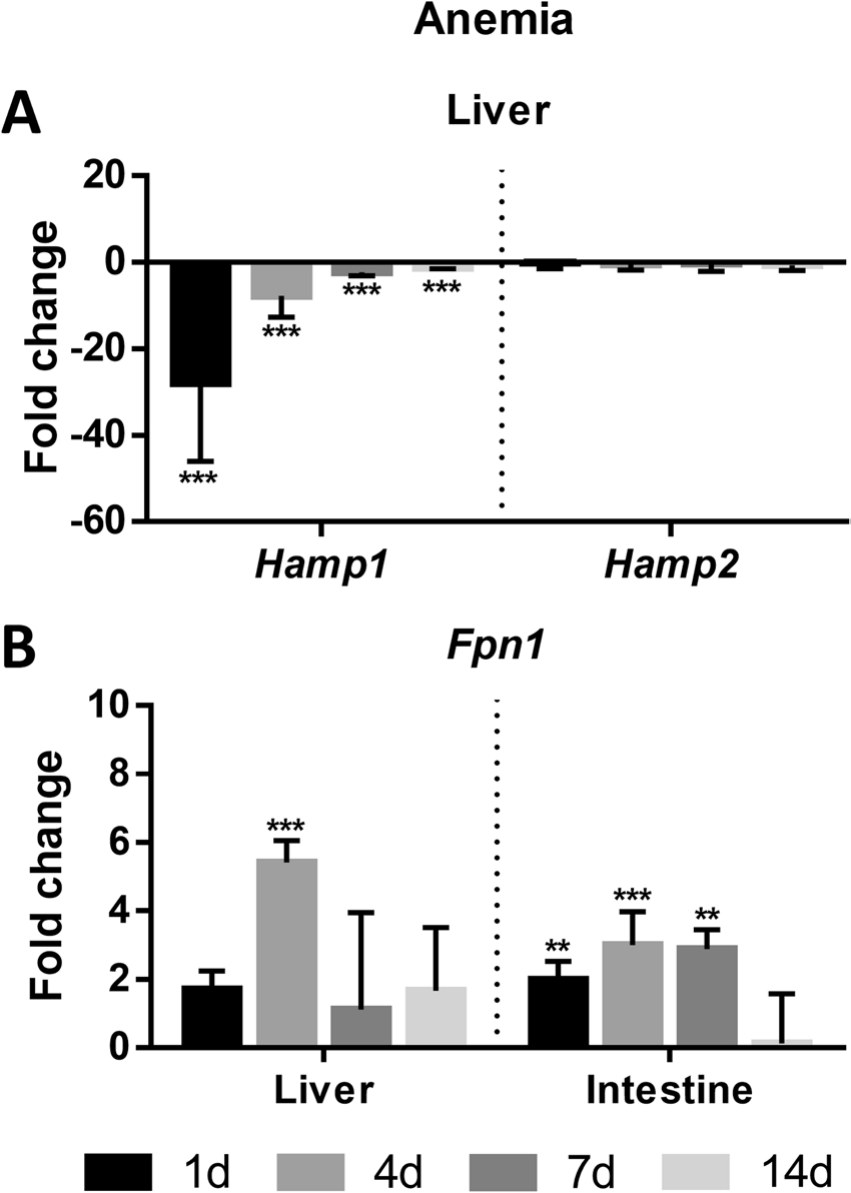
Sea bass (**A**) hepcidin expression in the liver and (**B**) ferroportin expression in the liver and intestine under experimental anemia. Fish were made anemic by removing 2% v/w body mass of blood through the caudal vessels (n = 4). Values are expressed as mean fold change ± SD. *Actb* was used as the housekeeping gene. Differences from the control groups were considered significant at *p < 0.05, **p < 0.01, and ***p < 0.001.

*Anemia*. *Hamp1* and *hamp2* expressions were evaluated in the liver (Fig. 5A), and *fpn1* expression was evaluated both in the liver and intestine (Fig. 5B) of anemic fish, 1, 4, 7, and 14 d after blood was drawn from the caudal vessels. *Hamp1* expression significantly decreased in the liver starting as early as day 1, with a gradual recovery towards control levels throughout the experiment, but never reaching them even at day 14. No significant changes were observed for *hamp2* at any experimental time point. A significant increase in *fpn1* expression was observed in the liver at day 4, with a recovery to control levels at day 7. In the intestine, *fpn1* expression gradually increased up to day 4, followed by a slight decrease at day 7 and recovering to control levels at day 14.

**Figure 6:**
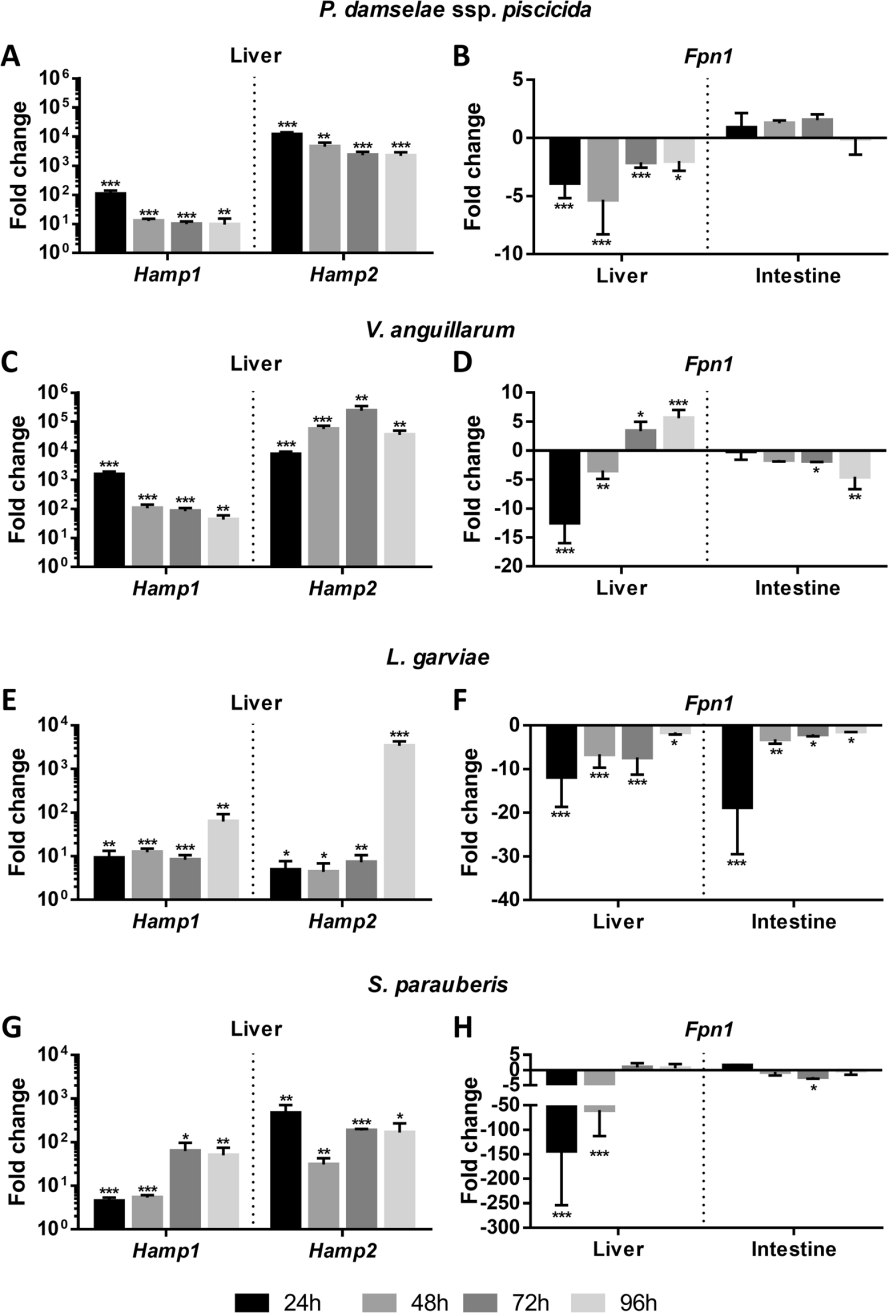
Sea bass *hamp1*, *hamp2* and *fpn1* expression after experimental infection with multiple pathogens. *Hamp1* and *hamp2* expressions were measured in the liver and *fpn1* expression was measured both in the liver and intestine, in fish i.p injected with 10^5^ CFU of *P*. *damselae* ssp. *piscicida* (**A** – *Hamp*, **B** – *Fpn1*), *V*. *anguillarum* (**C** – *Hamp*, **D** – *Fpn1*), *L*. *garviae* (**E** – *Hamp*, **F** – *Fpn1*) or *S*. *parauberis* (**G** – *Hamp*, **H** – *Fpn1*) (n = 4). Values are expressed as mean fold change ± SD. *Actb* was used as the housekeeping gene. Differences from the control groups were considered significant at *p < 0.05, **p < 0.01, and ***p < 0.001.

*Infection*. Number of CFU were determined in the spleen to confirm and evaluate the progression of the infections (sup. Figure 3). *Hamp1* and *hamp2* expressions were evaluated in the liver, and *fpn1* expression was evaluated both in the liver and intestine, 24, 48, 72 and 96 h after intraperitoneal infection with *P*. *damselae ssp*. *piscicida* (Fig. 6A,B), *V*. *anguillarum* (Fig. 6C,D), *L*. *garviae* (Fig. 6E,F) or *S. parauberis* (Fig. 6G,H). Overexpression was observed for both *hamp1* and *hamp2*, for all tested pathogens, albeit at much higher levels for *hamp2* when compared with *hamp1* and overall, with stronger responses elicited by Gram-negative bacteria. Changes in *fpn1* expression were variable depending on the pathogen, with an overall tendency for downregulation but with some exceptions. Infection with *P*. *damsela* induced a downregulation in the liver that lasted the duration of the experiment, but no significant changes were observed in the intestine. *V*. *anguillarum* produced the largest amplitude of variation in the liver, with significant downregulation of *fpn1* at 24 and 48 h and upregulation at 72 and 96 h, whereas in the intestine expression started decreasing at 72 h and reached minimum levels at 96 h. With *L*. *garviae*, similar patterns of *Fpn1* expression were observed in the liver and intestine, with the lowest levels observed at 24 h and a gradual recovery towards control levels throughout the duration of the experiment, but still significantly downregulated at 96 h. *S*. *parauberis* caused the most intense downregulation of *fpn1* in the liver among all tested bacteria, at 24 and 48 h, but values returned to normal levels at 72 h, whereas in the intestine, the only decrease in expression was observed at 72 h. Additionally, all tested bacteria led to decreases in hematocrit and circulating serum iron and an incremental accumulation of iron in the liver (sup. Figure 4).

### Effects of hepcidin administration on ferroportin regulation in sea bass

**Figure 7:**
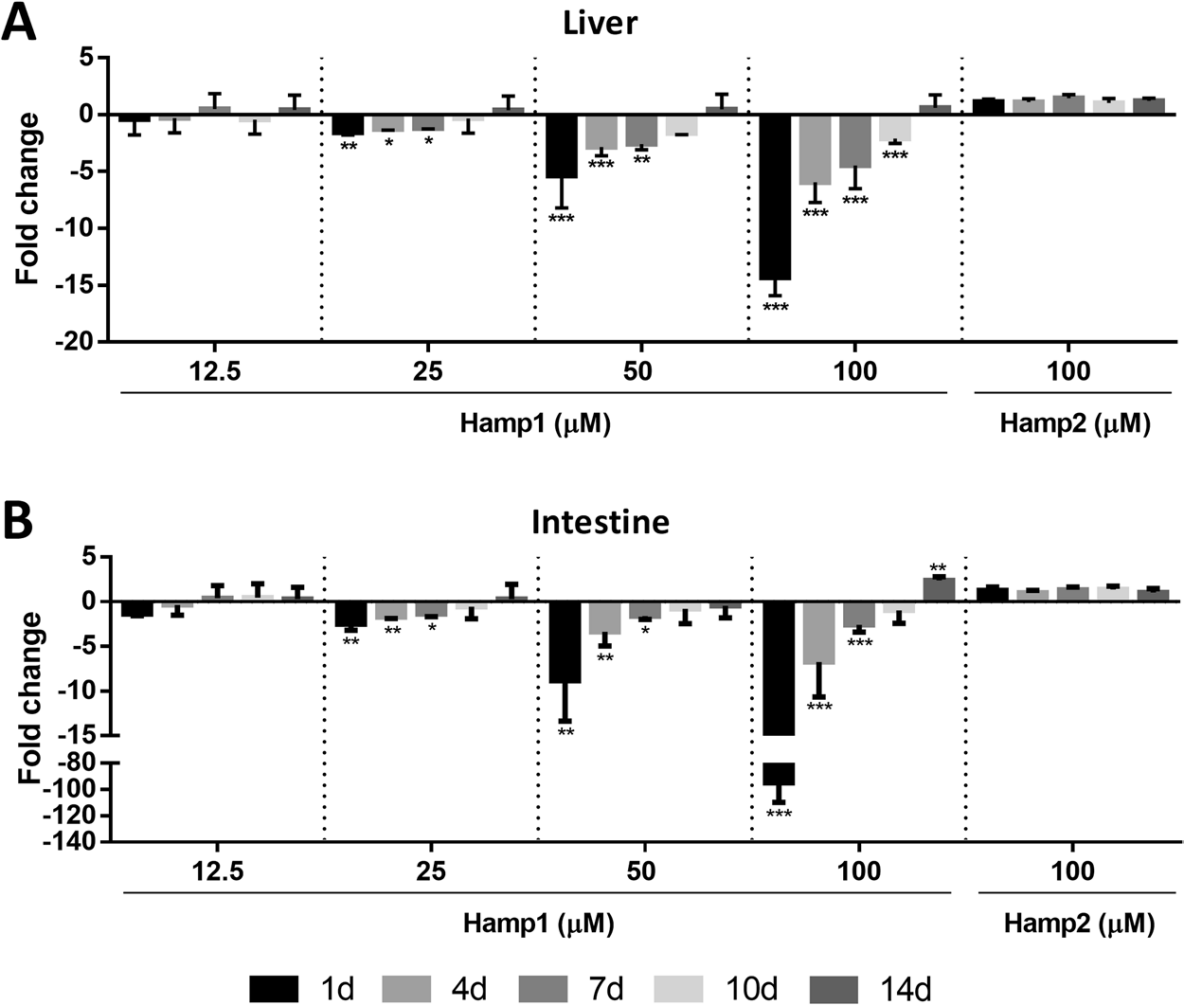
Sea bass fpn1 expression in the (**A**) liver and (**B**) intestine after peptide administration. Fish were i.p. injected with 100 µl of a 12.5, 25, 50 or 100 µM dilution of Hamp1 or Hamp2 synthetic peptide (n = 4). Values are expressed as mean fold change ± SD. *Actb* was used as the housekeeping gene. Differences from the control groups were considered significant at *p < 0.05, **p < 0.01, and ***p < 0.001.

Ferroportin expression was measured in the liver (Fig. 7A) and intestine (Fig. 7B) of fish injected with the synthetic mature peptides for sea bass hamp1-type and hamp2-type hepcidins. Hamp1 injection produced significant decreases of *fpn1* expression in both the liver and the intestine, in a dose dependent manner. The lowest concentration used did not elicit significant changes in *fpn1* expression in either organ, but starting at 25 µM, significant decreases were observed as soon as day 1, with gradual recovery throughout the experiment both in the liver and intestine. However, whereas in the liver *fpn1* expression gradually recovered to control levels up to day 14, in the intestine expression not only returned to control levels at day 10, but at the highest concentration of 100 µM exceeded them at day 14. The Hamp2 peptide failed to produce any significant changes in *fpn1* expression in the liver or the intestine, even at the highest concentration of 100 µM.

### Effects of sea bass Hepcidin administration on ferroportin regulation in murine derived macrophages

**Figure 8:**
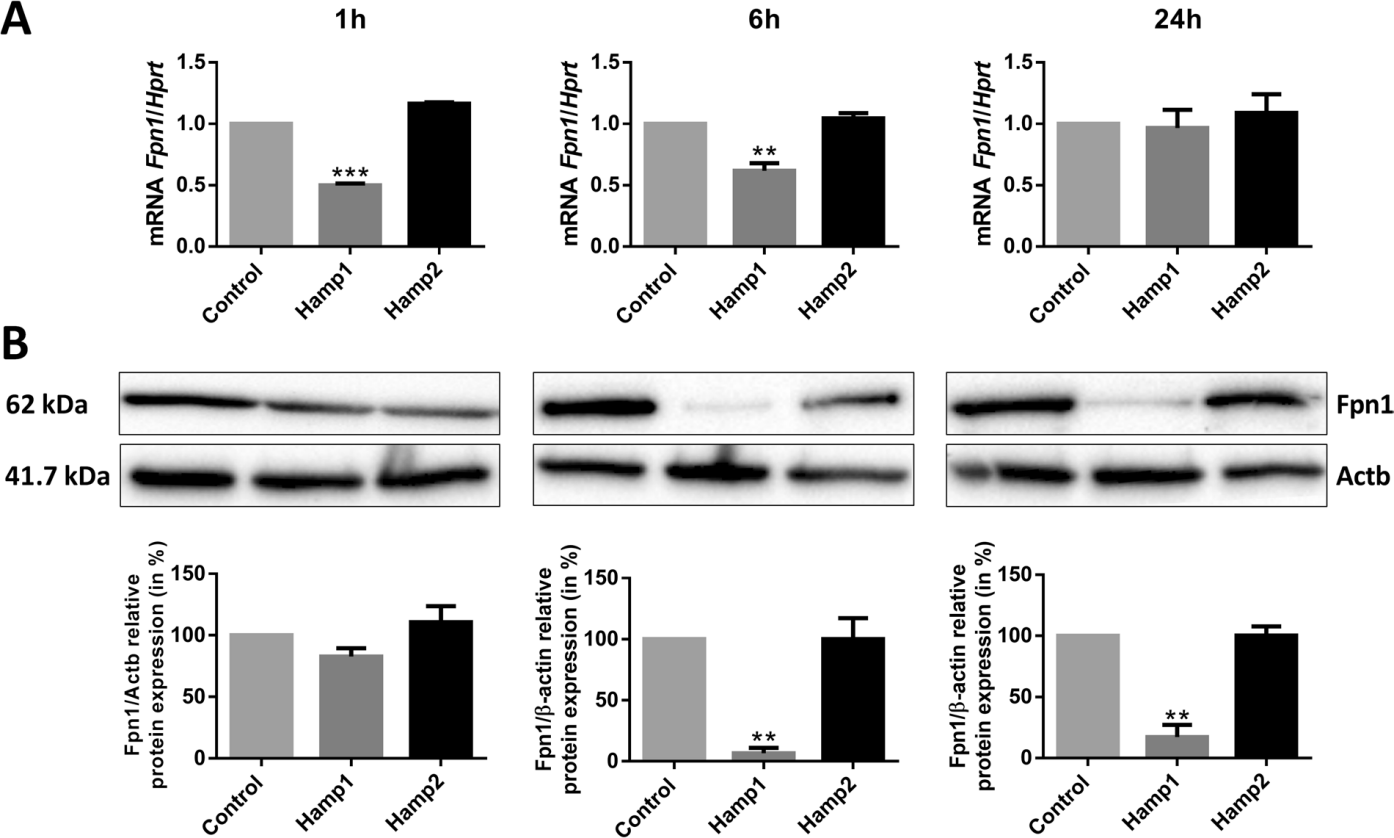
Effect of sea bass derived hepcidins on ferroportin from mouse macrophages. Mouse bone marrow-derived macrophages were treated with sea bass Hamp1 or Hamp2 peptides for 1, 6 and 24 hours, after which (**A**) *fpn1* mRNA expression and (**B**) Fpn1 protein levels were measured by real-time PCR and Western blot, respectively. Values are expressed as the means ± SD of three independent experiments. The Western blot images were obtained from 3 different blots (for 1, 6 and 24 hours) and each is representative of 3 independent experiments. Uncropped blots are presented in Supplementary Figure 5.

To determine if sea bass hepcidin peptides may have any effect on mammalian ferroportin, mouse bone marrow derived macrophages were treated with either sea bass Hamp1 or Hamp2 synthetic peptides for 1, 6 and 24 hours, and ferroportin was measured at the mRNA and protein levels. Treatment with Hamp1 caused significant decreases of *fpn1* mRNA expression starting as early as 1 h, with still lower than normal levels at 6 h and recovering to normal levels at 24 h (Fig. 8A). Similarly, a slight although not significant decrease in Fpn1 protein levels was observed at 1 h, with very significant decreases at both 6 and 24 h (Fig. 8B). Treatment with Hamp2 failed to produce any significant effects on either ferroportin mRNA expression or protein levels.

## Discussion

Ferroportin is the only known iron exporter in the cells^4–6^, playing a critical role in maintaining iron homeostasis. Its regulation is tightly connected with the antimicrobial peptide and key regulator of iron metabolism hepcidin, which causes its internalization and degradation, effectively blocking iron export from the cells^1,16^. However, whereas in mammals a single hepcidin gene exists (with the mouse being the sole known exception), many teleost fish have a large number of hepcidin genes that can be divided in two types, with markedly different functions^37^. As such, the study of the interaction between hepcidin and ferroportin in a teleost fish presenting two functional hepcidins can shed some light into its evolution and role, and may uncover novel connections with hepcidin not described in mammals.

In this study, we started by identifying and characterizing the ferroportin gene (*Fpn1*) in the European sea bass (*Dicentrarchus labrax*), a teleost fish presenting two already well characterized hepcidin types^37^. A single ferroportin transcript was found and characterized, encoding a putative 568 amino acids protein. Sequence alignment of the putative protein with ferroportins from other vertebrates showed that sea bass ferroportin also presents the characteristic features identified in human ferroportin: an extracellular hepcidin-binding loop with conserved key residues (Phe323, Cys325, Tyr332), critical for hepcidin binding to ferroportin^43,44^; an AP-2 adapter-binding motif, needed for clathrin-dependent endocytosis of ferroportin^44^; the cytoplasmic loop that undergoes hepcidin-dependent ubiquitination^14,44^. The latter is less conserved among the different vertebrates, but still shows a high degree of similarity among fish and a significant conservation of both termini in all species. Additionally, despite the lack of consensus as to the number of transmembrane domains present in ferroportin, which range from 8 to 12^4,6,19–24^, our study indicates the presence of twelve transmembrane domains in sea bass ferroportin, as described for human ferroportin^21,44^ and bacterial homologues^19,36^. Furthermore, a putative iron responsive element was also identified in the 5′ region of the *Fpn1* gene^10,11^. The presence of the mentioned features in sea bass ferroportin suggests that its function is mediated through mechanisms similar to the ones described for mammals.

Phylogenetic analysis of vertebrates ferroportin shows two different clusters, clearly separating fish from tetrapods and among fish, ferroportins can be further divided in four very closely related groups, with a high degree of evolutionary conservation.

Three dimensional molecular modeling of sea bass ferroportin was performed having as template the 3D structure of a ferroportin homologue from the bacterium *Bdellovibrio bacteriovirus* ^19,36^, as no suitable vertebrate structures could be found. In line with the predicted topology described for human^20,21^ and bacterial^19,36^ ferroportins, the 3D model of sea bass ferroportin presented 12 transmembrane (TM) helices, divided in two halves, one comprised of TM1 to TM6 and the other of TM7 to TM12, connected by a long cytosolic loop between TM6-TM7, and with both N- and C-termini being cytosolic. Just as with bacterial ferroportin^36^, the structure of TM7 consists of a longer helix (H7a) connected to a shorter helix (H7b) by a small unwound region. This was not observed for human ferroportin^20^, but modeling for that was based not on 3D structures of ferroportin homologues, but rather the structures of other members of the Major Facilitator Superfamily (MFS). The transmembrane regions predicted by protein comparison fall well within the helices obtained by 3D modeling, further reinforcing the idea of a total of 12 transmembranes for sea bass ferroportin.

Ferroportin mRNA was detected in all tested tissues. The highest basal expression was observed in the intestine, followed closely by the liver, with significant expressions also in the gill, spleen and head kidney. These findings are consistent with the known involvement of those tissues in iron metabolism. In fish, iron absorption occurs mostly in the mid and posterior regions of the intestine, where the iron importer Slc11a2 can be found^45^. The high constitutive levels of ferroportin observed in the posterior intestine further indicate this region as the major site for iron absorption in fish. The gill is also known to absorb small amounts of iron from the water, which is particularly important during the early stages of development^46–48^. However, the observed strong gill ferroportin expression in adults suggests a more significant role of this tissue on iron homeostasis. The liver is both the major organ of iron storage and sensor of changes in systemic iron requirements, as well as the major location for hepcidin expression in many fish^37^. The significant constitutive hepatic expression of ferroportin documented in this study reinforces the role of the liver as a regulator of iron levels, by increasing its release during conditions of limited iron availability (such as anemia, hypoxia, iron-poor diet) or blocking its export during conditions of iron overload or infection/inflammation. Finally, unlike mammals, where erythropoiesis is mostly performed in the bone marrow, in fish the spleen and head kidney assume that role, and ferroportin levels reflect the high numbers of reticuloendothelial macrophages present in these organs, known to be involved in iron recycling from senescent red blood cells^8^. A similar pattern of constitutive expression of ferroportin was also observed in another teleost fish, turbot (*Scophthalmus maximus*)^49^.

To better understand the interaction of hepcidin and ferroportin in a fish with two functional hepcidin types, their expression levels were evaluated in several experimental models, which included *in vivo* models of iron overload, anemia, infection with multiple pathogens and hepcidin administration. Due to the lack of sea bass anti-ferroportin antibodies, only variations in mRNA levels could be measured.

In response to the experimental iron overload, a massive increase in *hamp1* expression (but not *hamp2*) by the liver was accompanied by a decrease in *fpn1* expression both in the liver and in the intestine. The hepcidin response is consistent with what we previously observed and further reinforces the idea that Hamp1 in involved in iron homeostasis, whereas Hamp2 has little to no involvement in this process^37^. As a way to restore iron homeostasis, this huge increase in *hamp1* levels potentially leads to an increase in Hamp1 production, causing an inhibitory effect on *fpn1* expression, limiting iron release from the liver hepatocytes and intestinal enterocytes^1,9,16^, decreasing iron mobilization and absorption^45,50,51^.

In the experimentally anemic animals, the expression levels are opposite to what was observed during iron overload, with a large decrease of *hamp1* expression, and once again, no changes on *hamp2*. This was accompanied by a significant increase of *fpn1* expression in the liver and in the intestine, pointing for Hamp1 as the major regulator of ferroportin. Thus, during anemia, the fish iron-depleted status is detected by the liver, that responds by decreasing hepcidin production. The low hepcidin levels would in turn lead to a decreased ferroportin ubiquitination and degradation, allowing an increased iron mobilization through its release by the hepatocytes, reticuloendothelial macrophages and intestinal enterocytes^1,9,16^, and a compensatory increase of duodenal iron absorption^45,50,51^. Thus, the elevated *fpn1* expression will lead to increased absorption and mobilization of iron that may be made available to the hematopoietic organs in order to increase production of hemoglobin and erythrocytes, as previously shown in our laboratory^52^, to revert the artificially induced anemia.

In response to infection with Gram-negative and Gram-positive bacteria, increases in both *hamp1* and *hamp2* expression levels were observed, although usually higher for *hamp2*. We had previously proposed that the increases in *hamp1* expression are related to the need to increase iron retention and reduce iron mobilization, to limit availability for pathogens, whereas the increase in *hamp2* is related to an increased direct antimicrobial activity, according to the proposed different roles for each hepcidin type^37^, as attested by the present results and changes in hematocrit, serum iron and liver iron deposition (sup. Figure 4). All tested pathogens generally induced a decrease in *fpn1* expression both in the liver and intestine, with some exceptions. In the infection with *P*. *damsela*, *fpn1* expression was decreased in the liver, but no changes occurred in the intestine, possibly indicating that the liver response is sufficient to quell the infection without the need to limit dietary iron uptake. In the infection with *V*. *anguillarum* on the other hand, the decrease in *fpn1* expression observed in the liver in the first 48 hours was quickly replaced by a significant up-regulation towards the end of the experiment. Like most organisms, microbes need iron to multiply and different infectious pathogens use diverse strategies to obtain iron. Conversely, the host uses different counter-strategies to prevent its availability to pathogens^53^. For pathogens that develop in the bloodstream or extracellular spaces, the flux of iron released by ferroportin is advantageous. As such, an increased hepcidin production and associated *fpn1* inhibition would lead to a decreased iron release, limiting bacterial growth. However, intracellular pathogens would benefit from a decreased *fpn1* expression, since no iron is released from the infected cells. In this case, an opposite strategy would benefit the host, reducing hepcidin production and increasing *fpn1* expression, so intracellular iron would be released and the pathogens would be “starved”. For *V*. *anguillarum*, both mechanisms might be put in effect at different points during the course of the infection^54,55^, in order to maintain a steady supply of iron, as observed for other pathogens^56,57^. *V*. *anguillarum* has also shown to be resistant to hepcidin activity^37^, so this pathogen may be able to develop strategies to bypass hepcidin activity as an antimicrobial peptide, or as an iron regulator, thus reducing *fpn1* inhibition. Interestingly, another species of Vibrio, *V*. *vulnificus*, doesn’t seem to be able to bypass hepcidin activity, since it seems to be sensitive to hepcidin-mediated iron deprivation^58^.

We also evaluated the effects on *fpn1* expression after *in vivo* administration of either Hamp1 or Hamp2 synthetic peptides. Administration of Hamp1, previously shown to be mostly involved in the regulation of iron metabolism^37^, had a significant dose dependent effect on *fpn1* expression, particularly in the intestine, where expression was severely abrogated for the highest concentration used. This was accompanied by a slight but significant increase in liver iron content during the course of the experiment. These results make it clear that Hamp1 has an unequivocal inhibitory effect on *fpn1* mRNA expression *in vivo*. Interestingly, the modulation of *fpn1* expression levels by Hamp1 administration in turbot (*Scophthalmus maximu*) has been documented^49^. Administration of sea bass Hamp2 produced no effect on *fpn1* expression in the liver or the intestine, once again reaffirming the almost exclusively antimicrobial role of this type of hepcidin.

Finally, taking into account the high evolutionary conservation of ferroportin, as seen by the significant degree of homology and many shared features among teleosts and mammals, we evaluated the potential of sea bass hepcidins to interact with mammalian ferroportin, by treating mouse bone marrow-derived macrophages with our synthetic Hamp1 and Hamp2 peptides. Our data clearly shows that sea bass Hamp1 leads to profound decreases in mammalian ferroportin mRNA and protein expressions, whereas Hamp2 seems to have no effect either at the protein or mRNA levels, further confirming their differential roles as iron metabolism regulator and antimicrobial peptide, respectively. We can hypothesize that binding to ferroportin by hepcidin could signal for a decreased ferroportin mRNA expression, either at the transcriptional or post-transcriptional levels, but the exact molecular mechanisms for this are still mostly unknown. However, there is evidence for a variety of stimuli having an impact on ferroportin mRNA expression, including iron, hypoxia, anemia or infectious/inflammatory signals, mediated by hepcidin^5,37,52,59,60^, as well as by hepcidin-independent pathways^61^. The *in vivo* and *in vitro* results presented here clearly demonstrate the participation of Hamp1 peptide, but not Hamp2, in the transcriptional regulation of ferroportin, in organisms with two functional hepcidins. At the protein level, it has been shown that mammalian hepcidin (either human or mouse derived) leads to decreased ferroportin levels also in mammalian cells^9,12,62,63^ but it is the first time that the interaction between fish derived hepcidins and mammalian ferroportin is evaluated. These findings indicate that Fpn1 function is mediated by fish Hamp1 but not by Hamp2.

Taken together, these observations open an important road of research for the use of fish derived hepcidins, either prophylactically or therapeutically, not only in sea bass and other closely related fish species but also importantly in mammals, as different hepcidin types can be administered according to different needs: Hamp1-type hepcidins could be used to control iron metabolism and treat iron disorders, whereas Hamp2-type hepcidins could be used to prevent or fight a variety of infections, without directly interfering with the iron metabolism, contrary to mammalian hepcidins which are expected to exert both functions, potentially leading to undesired results such as anemia of inflammation.

In conclusion, we have isolated and functionally characterized ferroportin in the European sea bass and investigated its interaction with the two different hepcidin types, shedding some light on the evolution of hepcidin and ferroportin functional partnership in vertebrates. We have demonstrated that the role of ferroportin in a teleost fish with two distinct functional hepcidin types is mediated through the iron-regulator Hamp1 peptide, and not through the dedicated antimicrobial Hamp2, an interaction that occurs also with mammalian ferroportin, opening new pathways for the potential medical use of fish derived hepcidins.

## Materials and Methods

### Animals

Healthy European sea bass (*Dicentrarchus labrax*), with an average weight of 30 g, were provided by a commercial fish farm in the south of Portugal (Piscicultura do Vale da Lama, Lagos, Portugal). Prior to the experiments, fish were acclimated for 30 days to the fish holding facilities of the Instituto de Biologia Molecular e Celular (IBMC), Porto. Fish were kept in 500 liters recirculating sea water tanks at 20 ± 1 °C, with a 12-hour light/dark cycle and fed daily *ad libitum* with commercial fish feed with an iron content of approximately 200 mg iron/kg feed. Before each treatment, fish were anaesthetized with ethylene glycol monophenyl ether (2-phenoxyethanol, 0,3 ml/l, Merck, Algés, Portugal). All animal experiments were carried out in strict compliance with national and international animal use ethics guidelines, approved by the animal welfare and ethic committees of IBMC and conducted by experienced and trained FELASA Category C investigators.

### Isolation of sea bass ferroportin

Pairs of oligonucleotide PCR primers were designed according to conserved regions of *fpn1* mRNA sequences from other fish and mammalian species, available in the National Center for Biotechnology Information nucleotide database (http://www.ncbi.nlm.nih.gov/) and Ensembl (http://www.ensembl.org/) and cDNA preparations from liver and intestine were used in PCR amplifications. Both 5′ and 3′ RACE were carried out using the 5′/3′ RACE Kit, 2^nd^ Generation (Roche Applied Science, Amadora, Portugal) according to the manufacturer’s instructions. Conditions for PCR were: 94 °C for 2 min, 94 °C for 15 s, 60 °C for 30 s, 72 °C for 40 s, for 10 cycles; 94 °C for 15 s, 60 °C for 30 s, 72 °C for 40 s (plus 20 s/cycle), for 25 cycles, with a final elongation at 72 °C for 7 min. When necessary, a second PCR amplification was performed using these conditions for an additional 30 cycles. PCR products were run on 1.2% agarose gels, and then relevant fragments were purified with the Zymoclean Gel DNA Recovery Kit (Zymo Research, Irvine, CA), cloned into pCRII-TOPO vectors, propagated in One Shot Mach1-T1R Competent Cells (Thermo Fisher Scientific, Waltham MA, USA), and sent for sequencing (GATC Biotech, Konstanz, Germany). Both strands were sequenced, and chromatograms were analyzed in FinchTV (Geospiza, Seattle WA, USA) and assembled using Multalin (http://bioinfo.genopole-toulouse.prd.fr/multalin/multalin.html). Genomic DNA was isolated from sea bass red blood cells using the NZY Blood gDNA Isolation kit (NZYTech, Lisboa, Portugal), according to the manufacturer’s instructions; quantification was performed using a NanoDrop 1000 spectrophotometer (Thermo Fisher Scientific); quality was checked by agarose gel electrophoresis. One microgram of genomic DNA was amplified by RT-PCR with the primers based on the previously obtained cDNA sequences. Several PCR products were purified, cloned, and sent for sequencing as described earlier. Comparisons were made between cDNA and genomic DNA to assess the similarity of the coding regions and to identify intron/exon boundaries.

### Sequence alignment and phylogenetic analysis

Amino acid sequence alignments were performed using MUSCLE from MEGA6^64^. A phylogenetic tree was constructed using the Maximum Likelihood method, with the Jones–Taylor–Thornton (JTT) model, Nearest-Neighbor-Interchange heuristic model, complete deletion of gaps, and 10000 bootstrap replications. Sequences used for comparisons and phylogenetic trees and their accession numbers were as follows: *Oreochromis niloticus* (XP_003458401); *Gasterosteus aculeatus* (ENSGACT00000003573); *Fundulus heteroclitus* (XP_012706520); *Oryzias latipes* (XP_004081876); *Larimichthys crocea* (KKF14475); *Maylandia zebra* (XP_004559291); *Neolamprologus brichardi* (XP_006807681); *Stegastes partitus* (XP_008293299); *Notothenia coriiceps* (XP_010786941); *Cynoglossus semilaevis* (XP_008327232); *Tetraodon nigroviridis*(ENSTNIT00000021250); *Takifugu rubripes* (XP_011612001); *Salmo salar* (XP_014028507); *Oncorhynchus mykiss* (CDQ57307); *Danio rerio* (ADH03019); *Xenopus tropicalis* (ABL75285); *Gallus gallus* (NP_001012931); *Columba livia* (EMC79608); *Alligator sinensis* (XP_014381800); *Rattus norvegicus* (NP_579849); *Mus musculus* (NP_058613); *Homo sapiens* (NP_055400); *Caenorhabditis elegans* (CCD73436).

### Molecular modeling of sea bass ferroportin

Prediction of the three-dimensional structure of sea bass ferroportin was performed by protein homology detection/modeling, using the Phyre2 webserver^65^. Phyre2 improves on the original Phyre webserver, by scanning the profile and secondary structure prediction of the query sequence against a fold library using the alignment of hidden Markov models via HHPred/HHsearch^66^. To maximize sequence coverage and confidence, intensive modeling was selected. Obtained models were submitted to the SAVES metaserver (http://services.mbi.ucla.edu/SAVES/) to check and validate protein structures. 97.95% of the residues were found to be in the most favored and allowed regions of the Ramachandran plot. Molecular graphic images were obtained using the Polyview-3D webserver (http://polyview.cchmc.org/polyview3d.html)^67^.

### RNA isolation and cDNA synthesis

Total RNA was isolated from tissues with the PureLink RNA Mini Kit protocol for animal tissues (Thermo Fisher Scientific) with the optional on-column PureLink DNase treatment, according to the manufacturer’s instructions. Total RNA quantification was performed using a NanoDrop 1000 spectrophotometer (Thermo Fisher Scientific), and quality was assessed by running the samples in an Experion Automated Electrophoresis Station (Bio-Rad, Hercules, CA). For all samples, 1.25 µg of each were converted to cDNA by Thermoscript and an oligo(dT) 20 primer (Thermo Fisher Scientific), for 40 min at 50 °C, according to the manufacturer’s protocol.

### Constitutive expression of sea bass ferroportin

Five healthy sea bass were sacrificed, and several tissues were collected for RNA isolation and cDNA synthesis, as previously described. Relative levels of *fpn1* mRNA were quantified by real-time PCR analysis using an iQ5 Multicolor Real-Time PCR Detection System (Bio-Rad). A total of 1 μl of each cDNA sample was added to a reaction mix containing 10 μl iQ SYBR Green Supermix (Bio-Rad), 8.5 μl double distilled H_2_O, and 250 nM of each primer, making a total volume of 20 μl per reaction. A non-template control was included for each set of primers. The cycling profile was the following: 94 °C for 3.5 min, 40 cycles of 94 °C for 30 s, 59 °C for 30 s, and 72 °C for 30 s. Samples were prepared in duplicates, a melting curve was generated for every PCR product to confirm the specificity of the assays, and a dilution series was prepared to check the efficiency of the reactions. Beta actin (Actb) was used as the housekeeping gene. The comparative CT method (2^-ΔΔCT^ method) based on cycle threshold values was used to analyze gene expression levels.

### Sea bass *hamp1*, *hamp2* and *fpn1* mRNA expression in response to experimental iron overload, anemia or infection

To evaluate *hamp1*, *hamp2* and *fpn1* transcriptional regulations in response to different conditions, several experimental models were created, RNA was isolated and converted into cDNA, and gene expression analysis was conducted as previously described for the liver and intestine.

#### Iron overload

To induce iron overload, fish were intraperitoneally injected with 200 µl iron dextran (Sigma-Aldrich, St. Louis MO, USA) diluted in sterile PBS to a final concentration of 10 mg/ml, as previously reported^68^. Control fish were injected with 200 µl sterile PBS. At 1, 4, 7, 10 and 14 d after treatment, four fish from each of the experimental groups were anesthetized, and blood was drawn from the caudal vessels for evaluation of hematological parameters. Subsequently, fish were euthanized with an overdose of anesthetic and dissected; their tissues were excised, snap frozen in liquid nitrogen, and stored at −80 °C until further use.

#### Anemia

To induce the experimental anemias, fish were individually weighted and bled from the caudal vessels the equivalent v/w of 2% body mass (20 fish per group). Control fish were subjected to the same manipulation (anesthesia, weighting, pinching), but no blood was removed. At 1, 4, 7, and 14 d after treatment, four fish from each of the experimental groups were anesthetized, and blood was drawn from the caudal vessels for evaluation of hematological parameters. Subsequently, fish were euthanized with an overdose of anesthetic and dissected; their tissues were excised, snap frozen in liquid nitrogen, and stored at −80 °C until further use.

#### Infection

Two Gram-negative bacteria (*Photobacterium damselae* ssp. *piscicida*; *Vibrio anguillarum*) and two Gram-positive bacteria (*Lactococcus garviae*; *Streptococcus parauberis*) were used for the experimental infections. All bacteria were cultured to mid logarithmic growth in appropriate growth media, absorbance was measured at 600 nm and the bacteria were resuspended to a final concentration of 5.0 × 10^5^ CFUs ml^−1^. For the experimental infections, fish were anesthetized and i.p. injected with 200 µl (1.0 × 10^5^ CFU) of bacterial suspension. For the control group, fish were injected with 200 µl of sterile growth medium. At 24, 48, 72 and 96 h post infection, four fish from each group were anesthetized, and blood was drawn from the caudal vessels for evaluation of hematological parameters. Fish were then euthanized with an overdose of anesthetic and dissected; their tissues were excised, snap frozen in liquid nitrogen, and stored at −80 °C until further use. Mortality was assessed every 12 h during the course of the experimental infections and CFU counts in the spleen performed at every experimental time point.

### Biological effects of hepcidin peptides on ferroportin regulation *in vivo*

The biological effects of sea bass hepcidins on *fpn1* expression were evaluated. For that, synthetic peptides coding for the predicted mature peptides of Hamp1 (QSHLSLCRWCCNCCRGNKGCGFCCKF), and Hamp2 (HSSPGGCRFCCNCCPNMSGCGVCCRF) were commercially produced (Bachem AG, Bubendorf, Switzerland) and administered to healthy sea bass. Peptides were diluted in 1× PBS to final concentrations of 12.5, 25, 50 and 100 µM and each fish was i.p. injected with 100 µl. Control animals received a similar volume of saline. At 1, 4, 7, 10 and 14 d post peptide administration, four fish from each group were anesthetized, and blood was drawn from the caudal vessels for evaluation of hematological parameters. Fish were then euthanized with an overdose of anesthetic and dissected; their tissues were excised, snap frozen in liquid nitrogen, and stored at −80 °C until further use. Expression of *fpn1* was determined as previously described.

### Biological effects of sea bass derived hepcidins on mouse ferroportin *in vitro*

#### Murine Bone Marrow-derived Macrophages (BMM)

Macrophages were derived from the bone marrow of male BALB/c mice bred at the i3S animal facility. Each femur and tibia was flushed with Hank’s Balanced Salt Solution (HBSS, Gibco, Paisley, U.K.). The resulting cell suspension was centrifuged and re-suspended in Dulbecco’s Modified Eagle’s Medium (DMEM, Gibco, Paisley, U.K.) supplemented with 10 mM glutamine, 10 mM HEPES, 1 mM sodium pyruvate, 10% Fetal Bovine Serum (FBS, Gibco, USA) and 10% of L929 cell conditioned medium (LCCM) as a source of Macrophage Colony Stimulating Factor (M-CSF). Cells were cultured overnight at 37 °C in a 7% CO_2_ atmosphere to remove mature adherent cells. Non-adherent cells were collected with cold HBSS medium, washed and seeded at the concentration of 4 × 10^5^ cells/ml (3 ml/well, 6-well plates) and incubated at 37 °C in a 7% CO_2_ atmosphere. Four days after seeding, 10% of LCCM was added to the culture medium and on the 7^th^ and 10^th^ days, the medium was renewed. At day 10 of culture, when cells where fully differentiated into macrophages, 10 µl of Hamp1 and Hamp2 peptides were added to the wells, to a final concentration of 0.2 µM (0.6 µg/ml Hamp1, 0.55 µg/ml Hamp2), and left to incubate for 1, 6 and 24 hours, after which cells were harvested for RNA and protein extraction. *Fpn1 mRNA expression*. Media was discarded and 300 µl of lysis buffer (Life Sciences) containing 1% 2-Mercaptoethanol (Sigma Aldrich) were added to each well. Cells were then mixed with the lysis buffer and scrapped with the aid of a cell scrapper and the contents of three wells were collected to a centrifuge tube. RNA extraction was performed using the PureLink RNA Mini Kit protocol for animal cells (Thermo Fisher Scientific), according to the manufacturer’s instructions, converted into cDNA and expression of *fpn1* was determined as previously described. *Protein isolation*. Media from the wells was collected into centrifuge tubes and cells were harvested with PBS EDTA (5 mM) 15 min on ice, and collected to the same tube. They were then centrifuged at 1000x g for 10 min, washed with cold PBS and centrifuged again at 1000x g for 10 min. The cell pellet was resuspended in 30 µl of RIPA buffer (150 mM NaCl, 1.0% NP-40, 0.5% sodium deoxycholate, 0.1% SDS, 50 mM Tris, pH 8.0) and homogenized by 30 passages through a 26-gauge needle. Samples were stored at −80 °C until further used. *Western blot*. Cellular extracts were prepared in Laemmli buffer (from Bio-rad, Hercules, CA, USA). Equivalent amounts of protein (30 µg) were separated by electrophoresis in 10% SDS-polyacrylamide gels (SDS-PAGE) and electrophoretically transferred to a polyvinylidene difluoride (PVDF) membrane for 90 min at 100 V. After blocking the membrane with 5% BSA in TBST (50 mM Tris-HCl, pH 8; 154 mM NaCl; 0.1% Tween 20) for 1 h RT, membranes were incubated with the primary antibody following the manufacturer’s instructions: rabbit anti-FPN (Ferroportin/SLC40A1 Antibody from Novus, Littletown, CO, USA catalog #NPB1-21502), (1:300) 1 h RT, rabbit anti-beta-actin (Abcam, Cambridge, UK, catalog #8227), (1:5000) overnight 4 °C. Membranes were washed and incubated with the secondary anti-rabbit (1:10000) in 1% BSA in TBST. Membranes were then incubated with the Luminata Crescendo Western HRP substrate, imaged with ChemiDoc imaging system (Bio-Rad, Hercules, CA) and analysed in ImageLab software (Bio-Rad, Hercules, CA).

### Statistical analysis

Statistical analysis was carried out using GraphPad Prism 6 (GraphPad Software Inc, La Jolla CA, USA). Data normality was obtained by performing Kolmogorof-Smirnoff test and Student’s T-test was used for estimating statistical significance. Multiple comparisons were performed with One-way ANOVA and *post hoc* Student Newman-Keuls test. A p value < 0.05 was considered statistically significant.

### Data availability

All data generated or analysed during this study are included in this published article (and its Supplementary Information files).

## Electronic supplementary material

Supplementary Information
