# Supplementary material for: Hamp1 but not Hamp2 regulates ferroportin in fish with two functionally distinct hepcidin types
Source: Sci Rep. 2017 Nov 1;7:14793. doi: 10.1038/s41598-017-14933-5 (PMC5665920; doi:10.1038/s41598-017-14933-5)
Supplement: Supplementary file 1 — Supplementary Information [file 41598_2017_14933_MOESM1_ESM.pdf]

Hamp1 but not Hamp2 regulates ferroportin in fish with two functionally distinct hepcidin types

João V Neves[1,2,*], Miguel F Ramos[1,2], Ana C Moreira[1,2], Tânia Silva[1,2], Maria S Gomes[1,2,3], Pedro NS Rodrigues[1,2,3]

[1] i3S - Instituto de Investigação e Inovação em Saúde, Universidade do Porto, Portugal, Rua Alfredo Allen 208, 4200-135 Porto, Portugal

[2] IBMC – Instituto de Biologia Molecular e Celular, Universidade do Porto, Rua Alfredo Allen 208, 4200-135 Porto, Portugal

[3] ICBAS - Instituto de Ciências Biomédicas Abel Salazar, Universidade do Porto, Rua de Jorge Viterbo Ferreira 228, 4050-313 Porto, Portugal

* Corresponding author:

João V Neves

Iron and Innate Immunity

i3S - Instituto de Investigação e Inovação em Saúde, Rua Alfredo Allen 208, 4200-135 Porto, PORTUGAL

Tel: +351220408800

E-mail: jneves@ibmc.up.pt

```
                                                                                                  GTGTGTG      7
TGTGTGTGTGTGTGTGTGTGTGTGTGTGTGCGGTGGGAGGACACAATGTAAAATAGCGAGGGAGTCACGTGACTGCGTGGCTATATCCGAGG      97
CAGCGTAAACAGTGAGCAGAATGAGCGGAGGCAGCCGTCAGACGGACGGTGGCTGGATATTTCAGCCCTCACAGCTCGTGTCTTCTCTTT      187
TTTTCTTTCTTTACTGTTATTTCTTTCCGACTTCAGCTACAGTGATAGCTAAGTTTGGAGAGGAGGAAAGGAGGGAAGAACCCGCCGTGT      277
CTTTTTGTCTTTTGTTGTTTTTTTTTTTCCGCTGTTGTGTGTGTGGTGTCGCTGAGAGATAACAGTTAAGTGAAGACGTTTGCTTTAACG      367
TTGCATTATCAAAAAGAGGTAGGAACAACACTCACTTGCTGCTGGTTGTTTTTGTTTTTTTGAACCCATTTGGAAACAGCTTTTTAAAAA      457
ATGGATAACTCTGGAACTAAGAAGACGTGCATGGAATCCACCCGAGACTTCTTCACCTCGGCCAAATTTCTCATCTACATGGGCCACGCA      547
 M  D  N  S  G  T  K  K  T  C  M  E  S  T  R  D  F  F  T  S  A  K  F  L  I  Y  M  G  H  A   30
CTGTCAACCTGGGGCGACCGAATGTGGAACTTTGCCGTGGCCGTTTTCCTGGTGGAGCTGTACGGGAACAGCCTGCTGCTCACGGCCGTG      637
 L  S  T  W  G  D  R  M  W  N  F  A  V  A  V  F  L  V  E  L  Y  G  N  S  L  L  L  T  A  V   60
TACGGGCTGGTGGTGGCCGGCTCCGTGCTGCTGCTGGGGGCCATCATTGGGGACTGGGTGGACAAGAATCCCAGACTCAAAGTGGCCCAG      727
 Y  G  L  V  V  A  G  S  V  L  L  L  G  A  I  I  G  D  W  V  D  K  N  P  R  L  K  V  A  Q   90
ACTTCGCTGCTCGTCCAGAACAGTTGCGTCATCGTGTGTGGGATCCTCCTGATGGTTGTTTTCCAGTTCAAAGAACAGCTTGTGGAGCTT      817
 T  S  L  L  V  Q  N  S  C  V  I  V  C  G  I  L  L  M  V  V  F  Q  F  K  E  Q  L  V  E  L   120
TACAATGGATGGATTCTGACCACCTGCTACATTCTGGTGATCACCATCGCCAACATTGCCAACTTAGCCAGCACAGCTACATCCATCACC      907
 Y  N  G  W  I  L  T  T  C  Y  I  L  V  I  T  I  A  N  I  A  N  L  A  S  T  A  T  S  I  T   150
ATCCAAAGGGACTGGGTGGTGGTTGTGGCGGGTCAGGACAGCAGCAGGTTGGCAGATATGAATGCCACGGTGCGGATCATTGACCAGCTG      997
 I  Q  R  D  W  V  V  V  V  A  G  Q  D  S  S  R  L  A  D  M  N  A  T  V  R  I  I  D  Q  L   180
ACCAACATCTTGGCTCCTATGCTGGTGGGTCAGATAATGGCCTTCGGCTCCCATTTCATCGGCTGTGGGTTCATCTCTGGCTGGAACCTG      1087
 T  N  I  L  A  P  M  L  V  G  Q  I  M  A  F  G  S  H  F  I  G  C  G  F  I  S  G  W  N  L   210
TGCTCCATGTGTCTGGAGTACGCGCTGCTGTGGAAGGTCTACCAGAAGACGCCGGCGTTGGCCACGAAAGCTGGCCAGAAAGAGCAGCAG      1177
 C  S  M  C  L  E  Y  A  L  L  W  K  V  Y  Q  K  T  P  A  L  A  T  K  A  G  Q  K  E  Q  Q   240
CAGGAGCTCAAACAGCTCAGCCCCACGCGAGAGTTGGAGAACGGCCAGAGCCCTGAGGAATCGTCTCAGCCGCTCATGAATGAAACCTCA      1267
 Q  E  L  K  Q  L  S  P  T  R  E  L  E  N  G  Q  S  P  E  E  S  S  Q  P  L  M  N  E  T  S   270
GTCGTAACTAAGCCCGCCTCTCCCAAGCAGCGCGGCTGTTGCTATCAGGTGACCGAACCTCTGCGCACCTTCAAGGCAGGCTGGGTCGCT      1357
 V  V  T  K  P  A  S  P  K  Q  R  G  C  C  Y  Q  V  T  E  P  L  R  T  F  K  A  G  W  V  A   300
TACTACAACCAGAACATCTTCTTCGCCGGCATGTCCCTGGCTTTCCTCTACATGACGGTGCTGGGCTTCGACTGCATCACCACGGGCTAC      1447
 Y  Y  N  Q  N  I  F  F  A  G  M  S  L  A  F  L  Y  M  T  V  L  G  F  D  C  I  T  T  G  Y   330
GCCTACACGCAGGGGCTCAACGGCTCGGTGCTCAGCCTGCTGATGGGGGCCTCGGCCGTGTCGGGCATCTGCGGCACCGTCGCCTTCACC      1537
 A  Y  T  Q  G  L  N  G  S  V  L  S  L  L  M  G  A  S  A  V  S  G  I  C  G  T  V  A  F  T   360
TGGGTCCGCAAGAAGTGCGGCCTGATCCGCACAGGCTTCATCTCGGGCCTTGCCCAGCTGTCCTGCCTCATACTGTGTGTCGTCTCCGTC      1627
 W  V  R  K  K  C  G  L  I  R  T  G  F  I  S  G  L  A  Q  L  S  C  L  I  L  C  V  V  S  V   390
TTTGCTCCCGGGAGCCCCTTTGACCTCAGCGTCTCGCCCTTCCAGGACCTTTACACCCACCTGATCGGAGAGAAGCCGCTGCCCGAGGCC      1717
 F  A  P  G  S  P  F  D  L  S  V  S  P  F  Q  D  L  Y  T  H  L  I  G  E  K  P  L  P  E  A   420
GAGCACAGCCTCACCGGCGTTCTTACCAGTGGAAACGTCACTACTGCTGCACCAGCTGAAGAGCTGCCACCTCTGCAGTCCTACATGTCT      1807
 E  H  S  L  T  G  V  L  T  S  G  N  V  T  T  A  A  P  A  E  E  L  P  P  L  Q  S  Y  M  S   450
GTTTGTCTGCTGTTTGCTGGCGTCATTGCTGCTAGAGTTGGCCTGTGGTCTTTTGACCTGACCGTGACCCAGCTCATCCAGGAGAATGTG      1897
 V  C  L  L  F  A  G  V  I  A  A  R  V  G  L  W  S  F  D  L  T  V  T  Q  L  I  Q  E  N  V   480
ATCGAGTCGGAGCGAGGTGTGATCAACGGCGTCCAGAACTCCATGAATTACCTCTTAGATCTGCTGCACTTCATCATGGTGATACTGGCT      1987
 I  E  S  E  R  G  V  I  N  G  V  Q  N  S  M  N  Y  L  L  D  L  L  H  F  I  M  V  I  L  A   510
CCGAACCCGGAGGCCTTCGGCCTTCTGGTCATCATCTCTGTGTCCTTCGTGGCCATGGGTCACATTATGTACTTTCGGTTTGCCTACAAG      2077
 P  N  P  E  A  F  G  L  L  V  I  I  S  V  S  F  V  A  M  G  H  I  M  Y  F  R  F  A  Y  K   540
AGCCTGGGGAGCCGCCTCTTCCTATGCTGCTCGCCGGAGCAGAAGGTGGAGGAGGTAGACAACCCCTCACTTCCTACCACCGTCTAACCC      2167
 S  L  G  S  R  L  F  L  C  C  S  P  E  Q  K  V  E  E  V  D  N  P  S  L  P  T  T  V  *      568
CGTAAAGAGACTCTCTCCTGGGTACAAATCTCTCAAGCCTTACCCTTGCCCTGCATCTTATTTATTCTAACATCTTTTTTCTTTGTAGCT      2257
TGGCAGATGCTAATGCTAACCAGATAAGGCAACTTAACATGCTATGCGTAGCAATTAATAAAGCACCACTCATTATCAGAAAGCTTAAAT      2347
ACAGTTATAGAAAACTAATCCATAGCAAGACTTTAGGGGTAAGGCAGGGTTTCTCCTAACGTTTTCAGTCTGGGAGTCAGATGAGAGTCT      2437
CTCTTCTGTGGGTCCATGCTCTTTGATAGATGCTGTACTGTCGAACAAGGATCGCATCACTTCTAGGCTCGTGATCCCAATGTGGTGGTC      2527
CTGTTAAGACATCAGGGGAAATGGCGCTGTAAAATATTCTCCCAGAATGCCTCACTGCCTGGATGTAGCTGTAAAGATGTTTAAAAAGGA      2617
GATTCTCTTGCAGGGAAAGCAGGCACTGAGGCTTGGATTAATGGGGCGGCGCTGTCGTCTGAGGACCCTAAGAGACAGGCCGTCCCCCAA      2707
CTTTCCTCTCTTGACACTTGCCTCTCATTGGTGCTGTAGCTGTGTCCGCTAGCTGTTTCTTCAGCCTCGGCTGTGTGTCCTTTCTTGCAT      2797
ACATGTATGAATCGATCAGCTTTATCTTGTCGGTCGTCTTAATTAAGACTGGAGCGCTTTTAATTTTTTTATTTAATCAATATAAAAAAA      2887
AAAAAA                                                                                             2893
```

Supplementary Figure 1. DNA and predicted amino acid sequence of sea bass ferroportin. Nucleotides are indicated above and numbered to the right of each lane (upper row). The deducted amino acid is shown below the nucleotide sequence. Amino acids are indicated with italic letters and numbered to the right of each lane (lower row) ending with the TAA stop codon (represented by *). A predicted Iron Responsive Element (IRE) in the 5’ region is underlined.

```
                1                                                                                                                             120
D. labrax          MDNSGTK KTCMESTRDF FTSAKFLIYM GHALSTWGDR MWNFAVAVFL VELYGNSLLL TAVYGLVVAG SVLLLGAIIG DWVDKNPRLK VAQTSLLVQN SCVIVCGILL MVVFQFKEQL
H. sapiens      MTRAGDHNRQ RGCCGSLADY LTSAKFLLYL GHSLSTWGDR MWHFAVSVFL VELYGNSLLL TAVYGLVVAG SVLVLGAIIG DWVDKNARLK VAQTSLVVQN VSVILCGIIL MMVFLHKHEL
                                                                          #                        #  #

                121                                                                                                                           240
D. labrax       VELYNGWILT TCYILVITIA NIANLASTAT SITIQRDWVV VVAGQDSSRL ADMNATVRII DQLTNILAPM LVGQIMAFGS HFIGCGFISG WNLCSMCLEY ALLWKVYQKT PALATKAGQK
H. sapiens      LTMYHGWVLT SCYILIITIA NIANLASTAT AITIQRDWIV VVAGEDRSKL ANMNATIRRI DQLTNILAPM AVGQIMTFGS PVIGCGFISG WNLVSMCVEY VLLWKVYQKT PALAVKAGLK
                                         #             #  # ##       #    #        #

                241                                                                                                                           360
D. labrax       EQQQELKQLS PTRELENGQS PEESSQPLMN ETSVVTKPAS PKQRGCCYQV TEPLRTFKAG WVAYYNQNIF FAGMSLAFLY MTVLGFDCIT TGYAYTQGLN GSVLSLLMGA SAVSGICGTV
H. sapiens      EEETELKQLN LHKDTE--PK PLEGTHLMGV KDSNIHELEH EQEPTCASQM AEPFRTFRDG WVSYYNQPVF LAGMGLAFLY MTVLGFDCIT TGYAYTQGLS GSILSILMGA SAITGIMGTV
                       #                          #                                                         #  #

                361                                                                                                                           480
D. labrax       AFTWVRKKCG LIRTGFISGL AQLSCLILCV VSVFAPGSPF DLSVSPFQDL YTHLI-GEKP LPEAEHSLTG VLTSGNVTTA APA--EELPP LQSYMSVCLL FAGVIAARVG LWSFDLTVTQ
H. sapiens      AFTWLRRKCG LVRTGLISGL AQLSCLILCV ISVFMPGSPL DLSVSPFEDI RSRFIQGESI TPTKIPEITT EIYMSNGSNS ANIVPETSPE SVPIISVSLL FAGVIAARIG LWSFDLTVTQ
                                                  #

                481                                                                                                 574
D. labrax       LIQENVIESE RGVINGVQNS MNYLLDLLHF IMVILAPNPE AFGLLVIISV SFVAMGHIMY FRFAYKSLGS RLFLCCSPEQ KVEEVDNPSL PTTV
H. sapiens      LLQENVIESE RGIINGVQNS MNYLLDLLHF IMVILAPNPE AFGLLVLISV SFVAMGHIMY FRFAQNTLGN KLF-ACGPDA KEVRKENQAN TSVV
                            #
```

Supplementary Figure 2. A closer look at the amino acid alignment of sea bass and human ferroportin. Identical residues in red, different residues in black, gaps indicated by dashes. Signature features are indicated as follows: underline, transmembrane domains[21,44]; dotted underline, extracellular hepcidin-binding loop[43,44] with key residues required for hepcidin binding in bold; wavy underline, AP-2 adapter-binding motif for clathrin-dependent endocytosis[44]; hashtags (#) represent the positions of the most common and some uncommon polymorphisms of human ferroportin, which cause numerous of changes in ferroportin cellular localization, iron export activity (e.g. increased or decreased iron efflux) and interaction with hepcidin (e.g. hepcidin-sensitive or hepcidin-resistant): Y64N[30,32,33], A77D[21,22,31,32,33], G80S[32], N144D[30,32,33], N144H[21,22,28,30,31,32,33], N144T[22,32], D157G[21,25,28,32], ΔV162[21,22,25,28,31,32,33], L170F[22], N174I[32], Q182H[21,25,28,32], Q248H[30,31,32,33], D270V[32], G323V[21,25,28,32], C326S[32], C326Y[30,32,33], S388R[34], G490D[28,32,33]. All but one position (D270) are conserved not only between sea bass and human ferroportin, but between all ferroportins from the species presented in figure 1A.

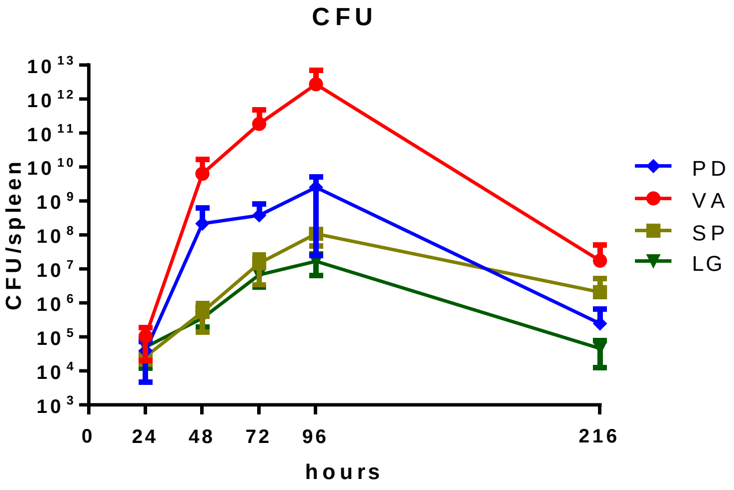

Supplementary Figure 3 – CFU counts in the spleen. Values are expressed as means ± S.D. (n=4). Counts were performed at 24, 48, 72, 96 and 216 hours post-infection. PD – *Photobacterium damsela* ssp *piscicida*, VA – *Vibrio anguillarum*, SP - *Streptococcus parauberis*, LG – *Lactococcus garvieae*.

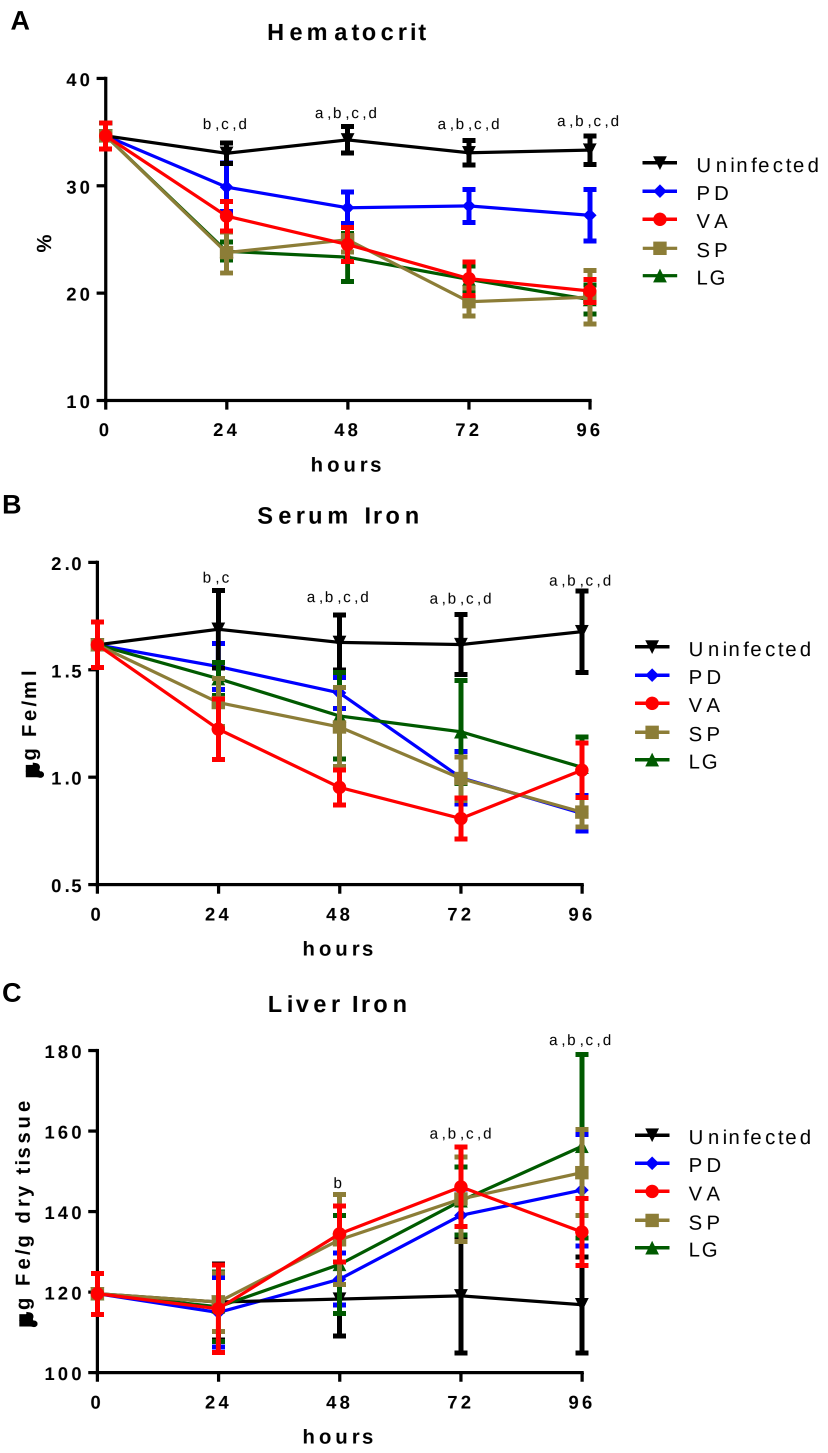

Supplementary Figure 4 - Hematological parameters and liver iron content in infected fish. (A) hematocrit, (B) serum iron and (C) liver iron. Values are expressed as means ± S.D. (n=4). Samples were collected at 24, 48, 72 and 96 hours post-infection. Untreated fish were used as a 0-day control (n=4). Differences from the controls were considered significant for $*p < 0.05$ as (a) PD – *Photobacterium damsela* ssp *piscicida*, (b) VA – *Vibrio anguillarum*, (c) SP - *Streptococcus parauberis* and (d) LG – *Lactococcus garvieae.*

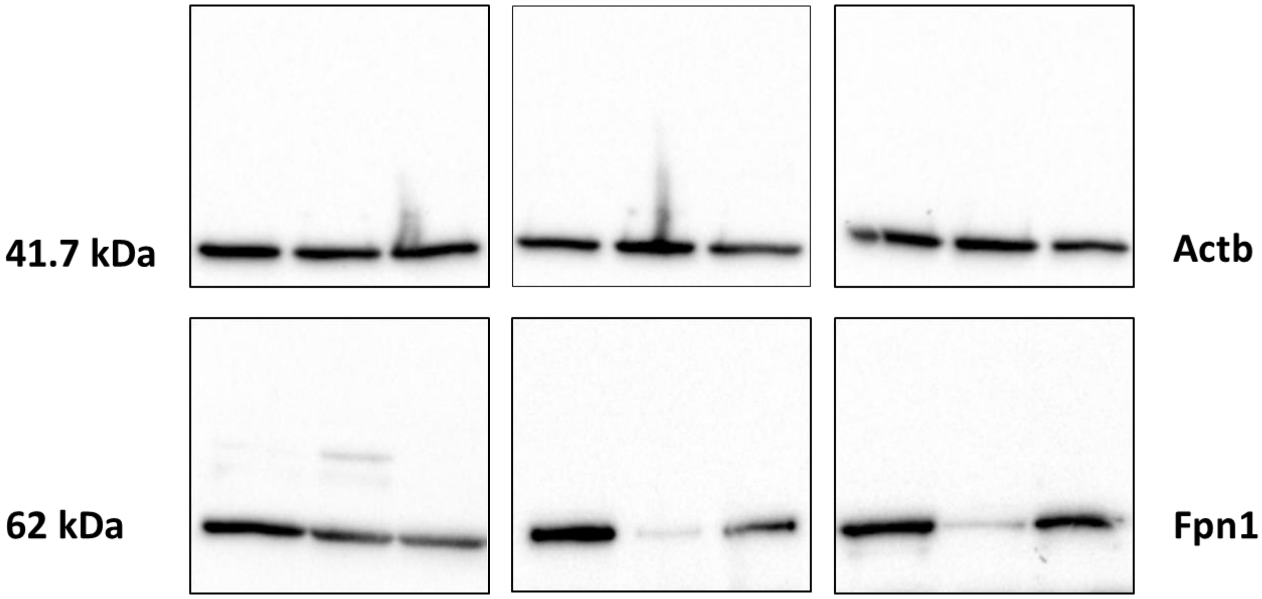

Supplementary Figure 5 - Uncropped Western blot membranes presented in cropped form in figure 8.

Supplementary Table 1 - Genomic comparison of sea bass ferroportin with other vertebrate and invertebrate species.

	Exon 1		Exon 2		Exon 3		Exon 4		Exon 5		Exon 6		Exon 7		Exon 8		
Dicentrarchus labrax	34	542	68	365	160	390	116	2132	127	310	246	122	639	442	317		
Gasterosteus aculeatus	43	282	68	363	160	241	116	1408	127	317	246	117	627	401	317		
Oreochromis niloticus	?	303	68	501	160	333	116	1030	127	638	246	?	?	848	323		
Oryzias latipes	?	35	56	382	160	208	116	1701	127	363	249	105	627	332	?		
Tetraodon nigroviridis	49	106	68	258	160	107	116	1206	127	869	246	113	636	227	317		
Takifugu rubripes	43	94	68	240	160	159	116	831	127	?	?	79	639	376	317		
Danio rerio	34	116	69	1790	160	192	116	1098	127	769	249	1168	621	1839	314		
Xenopus tropicalis	40	83	68	5325	160	555	116	2018	127	486	243	3541	657	1903	320		
Anolis carolinensis	49	7767	68	10341	160	2859	116	197	127	2115	246	1923	657	2907	320		
Gallus gallus	46	?	68	4167	160	829	116	271	127	3079	243	485	666	961	320		
Mus musculus	43	436	68	3401	160	2155	116	449	127	5827	246	705	639	1172	314		
Homo sapiens	43	537	68	4492	160	2199	116	1004	127	6115	246	1128	642	1392	314		
Caenorhabditis elegans	60	370	154	592	222	52	88	121	119	160	131	46	252	542	357	1322	147
	Exon 1		Exon 2		Exon 3		Exon 4		Exon 5		Exon 6		Exon 7		Exon 8		Exon 9

Among vertebrates, there is a high degree of conservation in both genomic structure, comprised by 8 exons and 7 introns, and exon sizes. Intron sizes are significantly variable, with tetrapods as usual presenting the bigger ones. Question marks (?) represent regions where available sequence was considered unreliable.
